# Cefazolin-induced hemolytic anemia: a case report and systematic review of literature

**DOI:** 10.1186/s40001-021-00604-9

**Published:** 2021-11-24

**Authors:** Elizabeth Mause, Mohammad Selim, Manasa Velagapudi

**Affiliations:** 1 Creighton University School of Medicine, Omaha, NE USA; 2 Department of Internal Medicine, Creighton University, Omaha, NE USA; 3 Department of Infectious Disease, Creighton University, Omaha, NE USA

**Keywords:** Cefazolin, Hemolytic anemia, Adverse drug reaction, Cefazolin allergy, Hemolysis, Eosinophilia

## Abstract

**Background:**

Cefazolin is a first-generation cephalosporin commonly used for skin and soft tissue infections, abdominal and orthopedic surgery prophylaxis, and methicillin-sensitive staph aureus. Cephalosporins as a whole are known potential inducers of hemolytic anemia; however, mechanism of action is primarily autoimmune, and compared to other drugs, cefazolin is the least common.

**Methods:**

A rare case report of cefazolin-induced hemolytic anemia “CIHA” and a systematic review of CIHA articles in English literature. Two authors performed review of publications and articles were selected based on inclusion and exclusion criteria. A systematic search of the literature yielded 768 entries with five case reports on cefazolin-induced hemolytic anemia.

**Case presentation/results:**

An 80-year-old female with methicillin-sensitive Staphylococcus aureus “MSSA” endocarditis. The patient was started on intravenous “IV” cefazolin that that resulted in hemolytic anemia and eosinophilia. Switching to vancomycin improved hemoglobin level and resolved eosinophilia. Four cefazolin-induced hemolytic anemia case reports and one population-based article with a case reported were analyzed with respect to direct antiglobulin test “DAT” (also known as the direct Coombs test) results, prior penicillin sensitivity, and acute anemia causes exclusion.

**Conclusions:**

CIHA is a rare cause of clinically significant anemia. The diagnosis of drug-induced anemia is one of exclusion. It is important to consider DAT results and prior penicillin sensitivity when evaluating a patient for cefazolin-induced hemolytic anemia. However, the frequency of cefazolin use and resultant anemia necessitates early recognition of hemolytic anemia and prompt discontinuation of cefazolin, especially with long-term use.

## Background

Hemolytic anemia has an extensive differential diagnosis. Common causes include autoimmune, drug-induced, or infections. The most common drug families include antibiotics, nonsteroidal anti-inflammatory drugs “NSAIDs”, and anti-cancer drugs. A 2007 article review identified 125 drugs evidenced to cause drug-induced hemolytic anemia “DIHA”. Cephalosporins were frequent culprits, especially cefotetan and ceftriaxone; however, a review article published in 2008 identified only two cases of cefazolin induced hemolytic anemia between 1971 and 2008 [1]. The present review focuses on case reports of cefazolin-induced hemolytic anemia with particular attention to the exclusion of other causes of anemia, direct antiglobulin test “DAT” results, and prior penicillin sensitivity. A review of articles from 1970 to 2021 revealed only a few case reports, and no other studies on CIHA were found. We also present an 80-year-old patient with cefazolin-induced hemolytic anemia diagnosed with CIHA and improved after cessation of the drug.

## Case presentation

An 80-year-old woman presented complaining of back pain and shortness of breath for 4 days. She had a past medical history of minor penicillin allergy, hypertension, hypothyroidism, gout, diastolic dysfunction, hyperlipidemia, transient ischemic attack, and non-ST-elevation myocardial infarction “NSTEMI.” Initial workup revealed elevated troponin and leukocytes/bacteria on urinalysis. Computed tomography angiogram “CTA” chest revealed bibasilar atelectasis/consolidation suspicious for pneumonia. The patient was diagnosed with severe sepsis secondary to bacterial pneumonia and urinary tract infection. She was started at an outside hospital on piperacillin–tazobactam, vancomycin, and azithromycin. The patient then developed acute anemia, and gastrointestinal bleeding was suspected. Esophagogastroduodenoscopy “EGD” was done and revealed a gastric ulcer with visible vessels that were treated. Hemoglobin had stabilized for days post-therapy. Then the patient was transferred to our tertiary center.

The first set of blood cultures came back positive for MSSA; antibiotics were downgraded to cefazolin. Magnetic resonance imaging “MRI” of the back was done given the patient’s bacteremia and back pain. Results demonstrated discitis and osteomyelitis at the level of T1–T2 vertebrae. Repeat blood cultures were positive again for MSSA. At this point, a transesophageal echocardiogram revealed mitral valve endocarditis. However, after visiting with the cardiothoracic surgeon, she decided to continue on medical treatment without surgical intervention.

Seven days after switching to cefazolin, the patient’s hemoglobin started to downtrend again and required multiple blood transfusions. Suspicion of recurrent gastric ulcer bleeding was high, but a repeated EGD revealed a healing gastric ulcer with no evidence of active bleeding. The patient’s workup for hemolytic anemia showed elevated lactate dehydrogenase “LDH,” indirect bilirubin levels, and low haptoglobin. DAT and complement testing yielded negative results. Peripheral blood smear showed schistocytes. Differential included side effects of one of the medications, shearing force from the endocarditis, or autoimmune hemolytic anemia “AIHA.” However, other bloodwork showed an up-trending of eosinophil count from the 5th-day post-switching to cefazolin. Presumptively, the patient was diagnosed with a possible allergic reaction to cefazolin resulting in CIHA. The diagnosis was in light of her prior known allergy to penicillin, declining hemoglobin, workup picture consistent with hemolytic anemia, the elevation of eosinophils after starting cefazolin, and the exclusion of other plausible causes of acute anemia. After switching to vancomycin, hemoglobin level gradually up-trended, and eosinophil count down-trended. The patient was discharged on a regimen of vancomycin for 6 weeks.

## Methods

A literature review was conducted on March 7, 2021, using the following databases: Academic Search Premier, JAMA Network, MEDLINE Complete, PubMed, Sage Journals, Science Direct, Wiley Online Library, and Springer Link. The language was restricted to English. There was no time restriction. The following search terms were used: cefazolin hemolytic anemia, cefazolin-induced hemolytic anemia, cefazolin drug-induced immune hemolytic anemia. Two authors reviewed publications. Articles were selected based on inclusion and exclusion criteria. The inclusion criteria were as follows: medical science journal articles, review articles, or research articles on:

1. Cefazolin-induced hemolytic anemia case reports
2. Literature reviews that mention cefazolin hemolytic anemia
3. Mechanism articles of cefazolin-induced hemolytic anemia.

Exclusion criteria included journal articles that only mention cephalosporins in general or other cephalosporins/beta-lactams antibiotic reactions (vs. cefazolin) or thrombocytopenic or neutropenic reactions to cefazolin (vs. anemia).

**Fig. 1:**
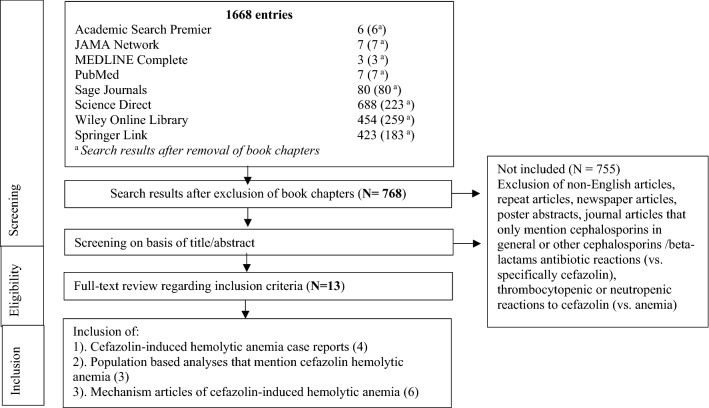
Systematic review of the literature for cefazolin induced hemolytic anemia

Disagreement between the two reviewers concerning the inclusion of particular studies was resolved. Selected studies were included for detailed analysis and data extraction. The parameters for data extraction were the following: exclusion of other causes of anemia, direct antiglobulin test “DAT” results, and prior penicillin sensitivity (Fig. 1).

A systematic review of the literature was conducted with progressive exclusion of entries. Exclusion was initially based on entry type (i.e., book chapters were excluded) and only full text, English language entries were included. Results were further stratified based on relevance and specificity for CIHA.

## Results

The systematic search yielded 768 entries after the removal of book chapters. After reviewing the titles and abstracts, 13 articles were included in the full-text review. Four case reports and one population-based article with a case reported were identified based on the inclusion criteria. The detailed selection process is shown in Fig. 1. We identified five reports of cefazolin-induced hemolytic anemia (Table 1).

**Table 1:** Cefazolin-induced anemia

Author (Year)	Study design	Presentation	Hemo-globin nadir	Penicillin sensitivity (Y/N)	DAT result ( ±)	Other anemia causes excluded (Y/N)	Outcome
Moake et al. [2]	Case report	39 yo F with right renal artery stenosis underwent a renal artery bypass with pre-operative and 4-day postoperative use of cefazolin	6.3	Y	+	Y	Complete resolution
Imam et al. [3]	Case report	70 yo M with acute anemia and elevated LDH post cefazolin administration; underwent mitral and tricuspid valve repair and Maze procedure	Δ3.0 g	NS	–	N ceftriaxone induced hemolytic anemia was also implicated	Complete resolution
Cerynik et al. [4]	Case report	50 yo M right total knee arthroplasty given pre-operative cefazolin developed anemia, then recovered; administration cefazolin 10 week postop due to knee swelling induced anemia again	14.9 6.9 Δ8	N	+	Y	Complete resolution
Macy, E. and Contreras, R. [5]	Retrospective population based analysis	37 yo F cardiac catheterization given pre-operative cefazolin with elevated lactate dehydrogenase post cefazolin administration	7.6	NS	NS	NS	Complete resolution
Moghaddam et al. [6]	Case report	24 yo F hysteroscopy for resection of the uterine septum	12.5 7.0 Δ5.5	N	+	N	The patient died of multiple organ failure

*NS  not specified, *Δ*  delta change

Table describing each study with reported cefazolin-induced hemolytic anemia. Characteristics of study design, patient presentation, hemoglobin lowest reported value, penicillin sensitivity, DAT result, and exclusion of other causes of anemia is specified for each case

Lowest hemoglobin value recorded and delta hemoglobin value was reported if available

In the 1978 study by Moake et al., a 39-year-old woman with a history of penicillin allergy received cefazolin prophylactically for renal artery bypass surgery [2]. Hemoglobin dropped on the 4th day, which prompted discontinuation of cefazolin with an improvement in the hemoglobin. A urinary tract infection “UTI” developed on the 6th day after the surgery. This prompted cephalothin administration, which resulted in the recurrence of the hemoglobin drop. Again, the patient had normalization of hemoglobin after discontinuation of cephalothin. Hemolytic anemia workup was positive, and she had positive IgG and complement components. Furthermore, the authors reported appropriate exclusion of other potential causes of anemia. The suspected mechanism was an interaction of cefazolin-coated red blood cells with preexisting anti-penicillin antibodies.

Another case was reported by Imam et al. published in 2007, of a 70-year-old man who had open-heart surgery for mitral and tricuspid valve repair and MAZE procedure for atrial fibrillation [3]. The patient’s recovery was complicated with Serratia pericarditis with bacteremia and pericardial effusion. The patient was discharged on daily ceftriaxone for 6 weeks, but he developed ceftriaxone-induced immune-mediated hemolytic anemia. We include this case report in our review, because the patient was on cefazolin for 2 day postoperative with a significant drop in hemoglobin count and a highly elevated lactate dehydrogenase level “LDH”. Acute blood loss anemia due to intra-operative or postoperative bleed was excluded, because the estimated blood loss during the surgery was 800 cc, and anemia developed 2 days after the surgery. Moreover, there were no apparent sources of blood loss in the few days post-surgery. The authors drew a direct connection between the later onset of ceftriaxone-induced hemolytic anemia and early exposure to cefazolin and considered it a CIHA.

Cerynik et al. reported a case of a 50-year-old man with hemolytic anemia secondary to cefazolin administration for right total knee arthroplasty [4]. Prophylactic cephalosporin was administered and postoperatively, IV cefazolin was continued, with anemia developing postoperative day 1. The patient received two units of packed red blood cells “PRBC” and was discharged home. He returned 10 weeks later with a knee swelling and was started empirically on IV cefazolin, after joint aspiration, with resultant hemolytic anemia. This prompted the discontinuation of cefazolin, which led to complete resolution of anemia. The underlying hemolytic anemia mechanism was suspected to be immune-mediated due to a rapid decrease in hemoglobin on the second exposure to cefazolin. Positive DAT, the presence of spherocytosis, and exclusion of other possible causes confirmed the diagnosis.

Macy, E. and Contreras, R. investigated the incidence of reported cephalosporin allergies among members of the Kaiser Permanente Southern California health plan between 2010 and 2012 via the electronic health record “EHR” [5]. In that analysis, the authors identified three possible cases of cephalosporin-induced hemolytic anemia. One of which involved cefazolin: a 37-year-old woman who underwent cardiac catheterization received cefazolin and was reported to have idiopathic hemolytic anemia that the authors suspected was likely cefazolin-induced. The patient recovered uneventfully over the next month. The authors of this review did not report workup for hemolytic anemia or exclusion of other possible causes.

In 2016, Moghaddam et al. reported a 24-year-old woman who developed hemolytic anemia after receiving pre-operative and postoperative cefazolin for a hysteroscopy [6]. Post-surgery anemia and hemoglobinuria were identified, and shortly after, disseminated intravascular coagulation “DIC” developed. She received several units of PRBCs and developed acute respiratory distress syndrome followed by complete anuria. The patient passed away from multi-organ failure. The authors hypothesized that preexisting antibodies were responsible for the hemolytic response due to rapid hemoglobin drop only a few hours after drug administration. The patient had no reported drug allergies; however, antibiotic use in livestock transmitted to meat products was cited as a potential exposure.

## Conclusions and discussion

We report a rare case of cefazolin-induced hemolytic anemia that developed 7 days after starting the medication. Markers of the hemolytic picture were positive. However, indicators of immune-mediated mechanisms such as DAT and complement were negative. The underlying mechanism of this hemolytic process was unclear. This prompted a revision of the English medical literature from 1970 till March 2021, in which we found only a handful of reported cases and articles on this topic.

DIHA is suspected in patients who present with anemia and hemolysis findings within days or weeks of exposure to a drug. Common mechanisms of DIHA include immune hemolysis or non-immune-mediated (oxidant injury). Naturally, with drug-induced immune hemolytic anemia “DIIHA”, DAT tends to be positive, with IgG or complement attached to the surface of red blood cells.

However, to our knowledge, a negative DAT does not definitively rule out DIIHA, because there are several drug-induced immune hemolytic anemia mechanisms [7]. First, the drug can act as a hapten—where antibodies are only detected in the presence of the drug [1, 4, 8]. Second, the drug can elicit an immune response mediated by autoantibodies—antibodies are drug-independent. Many penicillin and cephalosporin antibiotics such as cefazolin have been reported to cause hemolysis via the hapten reaction, where the drug is required for antibody binding [9].

We are suggesting that in our case, prior penicillin allergy might have played a role. The cross-reactivity between penicillins and cephalosporins can be attributed to similarities in their side chains [10]. Aminopenicillins (ampicillin) and aminocephalosporins (first generation—cefaclor, cefalexin, and cefadroxil) share an NH2 group at the R1 position and are susceptible to cross-reactivity [10]. Cefazolin (also a first-generation cephalosporin) does not have an NH2 group, making it less likely, but not impossible, for it to have cross-reactivity [11, 12]. Notably, cefazolin-induced hemolytic anemia is not always associated with a prior penicillin allergy. Besides our case, it was only reported in one out of the five case reports we reviewed.

In an attempt to understand the underlying mechanism of cefazolin-induced hemolysis, we have reviewed the better-studied mechanism of penicillin-induced hemolysis. The most accepted penicillin-induced hemolytic anemia mechanism involves penicillin-binding to RBC membrane proteins that antibodies (IgG usually) subsequently bind to, resulting in clearance via macrophages [1]. However, this exact mechanism is controversial for other antibiotics.^10^ In addition, the logistics of the second mechanism of drug-induced immune hemolytic anemia—autoantibody production—is still unknown [13]. There was no clear consensus among the five case reports about the mechanism of cefazolin-induced hemolytic anemia.

The first case has a clear link between the development of anemia and starting cefazolin with workup positive for immune-mediated hemolytic anemia. The perceived mechanism was an interaction of cefazolin-coated red blood cells with preexisting anti-penicillin antibodies.

The link is unclear in the second case, especially considering that the anemia started days after the surgery. At that time, no workup for hemolytic anemia was done nor exclusion of other possible causes. The diagnosis of CIHA was more theoretical based on the later ceftriaxone-induced immune-mediated hemolytic anemia.

The third case represents a strong link between anemia development and cefazolin exposure. The patient was diagnosed with immune-mediated hemolytic anemia.

The fourth case is a retrograde data collection with no clear evidence that the anemia was secondary to hemolysis nor immune-mediated. The authors of the article suspected a link to cefazolin but did not establish clear causation or speculate on the mechanism.

In the fifth case, although DAT was strongly positive, the diagnosis of cefazolin-induced immune hemolytic anemia is not convincing. The author’s conducted several lab experiments to exclude other possible causes of hemolysis, but the patient developed DIC post major surgery. Many studies indicate that hemolytic anemia might be an early sign of DIC; however, there is a lack of evidence that this development was linked to cefazolin.

CIHA is a rare cause of clinically significant anemia. Five case reports of CIHA exist in the literature with varying degrees of DAT reactivity. Mechanism of action in three of five is most consistent with an immune-mediated process. Although the prevalence and molecular mechanism of CIHA are not well understood, the frequency of cefazolin use and resultant potentially severe anemia necessitates a high degree of clinical suspicion for early recognition of hemolytic anemia and prompt discontinuation of cefazolin.

## Data Availability

Not applicable.

## Abbreviations

CIHA: Cefazolin-induced hemolytic anemia
MSSA: Methicillin-sensitive Staphylococcus aureus
IV: Intravenous
DAT: Direct antiglobulin test
NSAIDs: Nonsteroidal anti-inflammatory drugs
DIHA: Drug-induced hemolytic anemia
NSTEMI: Non-ST-elevation myocardial infarction
CTA: Computed tomography angiogram
EGD: Esophagogastroduodenoscopy
MRI: Magnetic resonance imaging
LDH: Lactate dehydrogenase
AIHA: Autoimmune hemolytic anemia
UTI: Urinary tract infection
PRBC: Packed red blood cells
EHR: Electronic health record
DIC: Disseminated intravascular coagulation
DIIHA: Drug-induced immune hemolytic anemia

## References

[1] Garratty G (2009). Drug-induced immune hemolytic anemia. Hematology.

[2] Moake JL (1978). Hemolysis induced by cefazolin and cephalothin in a patient with penicillin sensitivity. Transfusion.

[3] Imam SN, Wright K, Bhoopalam N, Choudhury A (2008). Hemolytic anemia from ceftriaxone in an elderly patient: a case report. J Am Med Dir Assoc.

[4] Cerynik DL, Lee G, Fayssoux R, Amin NH (2007). Case report: cefazolin-induced hemolytic anemia. Clin Orthop Relat Res.

[5] Macy E, Contreras R (2014). Adverse reactions associated with oral and parenteral use of cephalosporins: a retrospective population-based analysis. J Allergy Clin Immunol.

[6] Moghaddam M, Razzaghi F, Sheibani H, Pourfathollah AA (2016). A fatal case of cefazolin-induced immune hemolytic anemia in Iran. J Clin Exp Pathol.

[7] Johnson ST, Fueger JT, Gottschall JL (2007). One center’s experience: the serology and drugs associated with drug-induced immune hemolytic anemia—a new paradigm. Transfusion.

[8] Johnson ST (2009). Drug-induced immune hemolytic anemia. Transfus Apheres Sci.

[9] Mine Y, Nishida M, Goto S, Kuwahara S (1970). Studies on direct coombs reaction by cefazolin in vitro. J Antibiot.

[10] Wurpts G, Aberer W, Dickel H (2019). Guideline on diagnostic procedures for suspected hypersensitivity to beta-lactam antibiotics. Allergo J Int.

[11] Shenoy ES, Macy E, Rowe T, Blumenthal KG (2019). Evaluation and management of penicillin allergy: a review. JAMA.

[12] Pipet A, Veyrac G, Wessel F (2011). A statement on cefazolin immediate hypersensitivity: data from a large database, and focus on the cross-reactivities. Clin Exp Allergy.

[13] Garraty G (2010). Immune hemolytic anemia associated with drug therapy. Blood Rev.

